# The Multivalent Role of Fibronectin-Binding Proteins A and B (FnBPA and FnBPB) of *Staphylococcus aureus* in Host Infections

**DOI:** 10.3389/fmicb.2020.02054

**Published:** 2020-08-26

**Authors:** Pietro Speziale, Giampiero Pietrocola

**Affiliations:** Department of Molecular Medicine, Unit of Biochemistry, University of Pavia, Pavia, Italy

**Keywords:** *Staphylococcus aureus*, FnBPA, FnBPB, adhesin, invasin, extracellular matrix protein, binding mechanism, virulence factor

## Abstract

*Staphylococcus aureus*, one of the most important human pathogens, is the causative agent of several infectious diseases including sepsis, pneumonia, osteomyelitis, endocarditis and soft tissue infections. This pathogenicity is due to a multitude of virulence factors including several cell wall-anchored proteins (CWA). CWA proteins have modular structures with distinct domains binding different ligands. The majority of *S. aureus* strains express two CWA fibronectin (Fn)-binding adhesins FnBPA and FnBPB (Fn-binding proteins A and B), which are encoded by closely related genes. The N-terminus of FnBPA and FnBPB comprises an A domain which binds ligands such as fibrinogen, elastin and plasminogen. The A domain of FnBPB also interacts with histones and this binding results in the neutralization of the antimicrobial activity of these molecules. The C-terminal moiety of these adhesins comprises a long, intrinsically disordered domain composed of 11/10 fibronectin-binding repeats. These repetitive motifs of FnBPs promote invasion of cells that are not usually phagocytic *via* a mechanism by which they interact with integrin α_5_β_1_ through a Fn mediated-bridge. The FnBPA and FnBPB A domains engage in homophilic cell-cell interactions and promote biofilm formation and enhance platelet aggregation. In this review we update the current understanding of the structure and functional properties of FnBPs and emphasize the role they may have in the staphylococcal infections.

## Introduction

*Staphylococcus aureus*, a formidable pathogen that colonizes 30% of the population asymptomatically (Wertheim et al., 2005), is a major etiological agent of soft tissue infections such as cellulitis and superficial skin disease and a serious cause of abscesses, sepsis, pneumonia and endocarditis (Tong et al., 2015). In addition, to having increasing antibiotic resistance, the bacterium is a master in adapting to its host by avoiding almost every facet of the immune system (de Jong et al., 2019). *S. aureus* can express a broad multitude of virulence factors, most of which are surface proteins covalently anchored to wall peptidoglycan (CWA, cell wall-anchored proteins). The most studied group of this family of proteins is represented by the microbial surface components recognizing adhesive matrix molecules (MSCRAMMs). All MSCRAMMs share a similar structural organization and are involved in binding specific host ligands (Foster et al., 2014). The fibronectin (Fn)-binding MSCRAMMs FnBPA and FnBPB (fibronectin-binding protein A and B) are among the most intensively studied proteins of *S. aureus*. Since the initial discovery of the Fn binding by *S. aureus* (Kuusela, 1978) and identification of FnBPs (Rydén et al., 1983; Jönsson et al., 1991), studies have been focussed on their biochemical characterization and the mechanism of binding to Fn (Schwarz-Linek et al., 2003; Meenan et al., 2007; Bingham et al., 2008) and fibrinogen (Fbg) (Wann et al., 2000; Stemberk et al., 2014). FnBPs were found to be involved in invasion of a variety of several non-phagocytic cell lines (Sinha et al., 1999; Liang et al., 2016; Prystopiuk et al., 2018). Furthermore, FnBPs were shown to bind to elastin (Roche et al., 2004), plasminogen (Plg) (Pietrocola et al., 2016) and histones (Pietrocola et al., 2019) and to have an important role in the formation of biofilm (O’Neill et al., 2008; Geoghegan et al., 2013; Speziale et al., 2014; Herman-Bausier et al., 2015). This multifactorial capacity to bind several ligands is closely related to the pathogenicity of the bacterium. In this review we focus on the structural and functional aspects of FnBPs.

## Regulation of FnBPs Expression

FnBPs, as with many other MSCRAMMs, are predominantly present at a detectable and functional level on the surface of *S. aureus* cells during the exponential phase of growth. This suggests that the transcription of the *fnb* genes is prevalent in exponential phase and that FnBPA and FnBPB may be degraded by protease and be shed in the medium during stationary phase. However, during the stationary phase a sufficient amount of protein remains on the surface of the bacterial cells, as indicated by the ability of bacteria to retain binding to Fn. The *fnb* genes are subjected to control at the transcription level by Agr (accessory gene regulator) and Sar (Staphylococcal accessory regulator) global regulators (Cheung et al., 1992; Novick et al., 1993; Greene et al., 1995; Wolz et al., 2000). The Sar protein activates the synthesis of FnBPs during the exponential phase (Cheung et al., 1992; Giraudo et al., 1997), while Agr down-regulates transcription of genes for Fn-binding proteins post-exponentially (Saravia-Otten et al., 1997).

## FnBPs Structure and Organization

Fn-binding proteins FnBPA and FnBPB are encoded by closely related genes *fnbA* and *fnbB* (Signäs et al., 1989; Jönsson et al., 1991). Analysis of a collection of 163 clinical isolates revealed that the majority of *S. aureus* strains contain both the genes and that some strains, notably those from CC30 and CC45 lineages, do not express FnBPB (for example, the epidemic health care-associated MRSA (HA-MRSA) strain 252) (Peacock et al., 2000; McCarthy and Lindsay, 2010). Both the proteins comprise an N-terminal signal sequence and a sorting signal including a Leu-Pro-X-Thr-Gly (where X stands for any amino acid) motif, a hydrophobic membrane region and a cytosolic tail, at the C-terminus. The most promiscuous ligand-binding region of FnBPA and FnBPB resides in the N-terminal A domain, which is composed of three folded IgG-like subdomains (from N1 to N3). The combination of N2 and N3 subdomains forms a hydrophobic groove that binds ligands. Located between the A domain and the wall spanning region is a long, unstructured Fn-binding repetitive region (FnBR) composed of 11 repeats in FnBPA and 10 in FnBPB.

**FIGURE 1:**
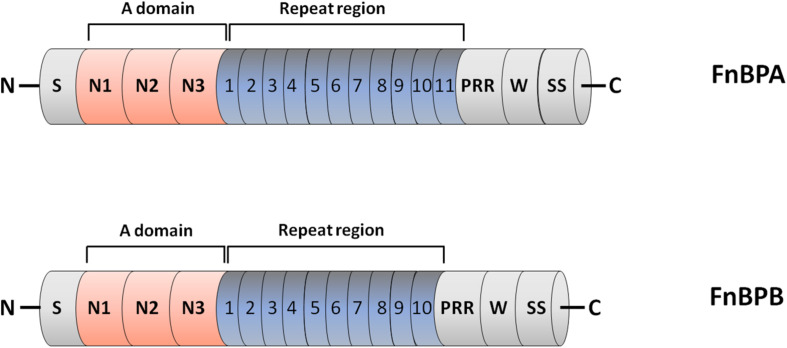
Domain organization of fibronectin-binding protein A and B (FnBPA and FnBPB). The primary translation products of FnBPA and FnBPB proteins contain a signal sequence (S) at the N-terminus and a sorting signal (SS) at the C-terminus. The N-terminal A region of both proteins contains three separately folded subdomains, termed N1, N2 and N3. N2 and N3 form IgG-like folds that combined bind ligands such as fibrinogen by the DLL mechanism. Located distal to the A domain is an unstructured region consisting of tandemly arranged motifs (11 in FnBPA and 10 in FnBPB) that individually bind to the N-terminal domain of fibronectin. Also shown are the locations of proline rich region (PRR) and wall spanning region (W).

The Fn-binding repeats bind individually to the N-terminal domain (NTD) of Fn and have considerable sequence variations, which in turn explains the differences in affinity of each repeat for the ligand (Figure 1).

## Ligands of FnBPA/FnBPB

FnBPA and FnBPB are good examples of promiscuous proteins that exhibit a great capability to interact with several structurally different host extracellular matrix/plasma proteins such as Fbg, Fn and elastin. Recently, these proteins have been shown to bind Plg/plasmin, a blood component that degrades many blood plasma proteins including fibrin clots, and histones.

### Fibronectin

Fn is a large glycoprotein composed of two almost identical subunits. It occurs in a soluble form in plasma and other biological fluids and in a fibrillar or reticular multimeric form in the extracellular matrix (ECM), an organized network of secreted macromolecules immobilized in the extracellular space. Functionally, Fn is involved in attachment of cells to surfaces, assembly of ECM, angiogenesis and would repair. The transcript of Fn gene generates by alternative splicing several mRNAs, each encoding similar but not identical monomeric subunits (Henderson et al., 2011).

The subunits of the dimer of both plasma and cellular Fn are disulfide-bonded at the extreme C-terminal ends. The monomers are composed of three different types of modules named type I, II and III repeated units. Sets of adjacent modules in both the chains form specific domains, each involved in binding to various partners (proteins and carbohydrates).

The N-terminal domain (NTD) interacts with ligands such as fibrin, thrombospondin-1, and heparin and many bacterial adhesins, including FnBPA and FnBPB, and is the major site for Fn self-association in the matrix. The NTD contains five type I modules, each of which consists of a double- and a triple-stranded antiparallel β-sheets stacked on top of each other. Each module has four cys residues, which form disulfide bridges that stabilize a double loop structure (Potts et al., 1999; Rudiño-Piñera et al., 2007).

Downstream to this region is the gelatin/collagen-binding domain composed of four type I and two type II modules that also binds to tissue transglutaminase. The type II modules (approximately 45 residues each) consist of two double-stranded anti-parallel β-sheets connected by a loop and are stabilized by two intrachain dsulfide bridges (Sticht et al., 1998). All modules contribute to the optimal interaction with gelatin/collagen (Katagiri et al., 2003).

**FIGURE 2:**
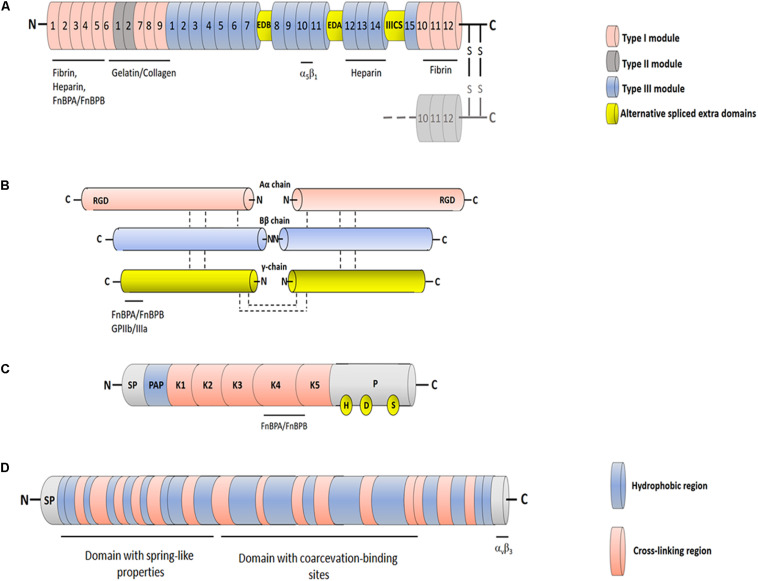
Schematic representation of FnBPs ligands. **(A)** Fibronectin. Each Fn chain consists of three different types of modules with distinct folds: FnI, FnII and FnIII. Sets of adjacent modules form functional protease-stable domains, including the N-terminal domain, the gelatin-binding domain and the cell-binding domain. The cell-binding domain harbors the RGD motif within the 10th FnIII module and “synergy site” in the 9th FnIII module, both involved in interaction with integrin α_5_β_1_. Cellular Fn includes two variable proportions of alternatively spliced FnIII modules EDB and EDA (extracellular domains B and A) and one connecting segment (IIICS). Binding sites for different binding partners are indicated. **(B)** Fibrinogen. Fbg is composed of three pairs of non-identical chains named Aα, Bβ and γ. The three chains are connected by disulfide bonds and are in hand to hand conformation forming a central E domain from which fibrinopetides A and B are released by the action of thrombin. The E domain is connected to the C-terminal D domains by coiled-coil region. The C-terminus of the γ chain includes the binding sites for integrin GPIIb/IIIa and FnBPA and FnFPB. **(C)** Plasminogen. Circulating Plg comprises at the N-terminal end a signal peptide (SP) and a preactivation region (PAP) followed by five consecutive disulphide-bonded kringle domains (K1-K5) and a serine protease domain (P). The K4 domain is supposed to be the binding site for FnBPs. The catalytic triad in the protease domain formed by residues His^603^, Asp^646^ and Ser^741^ is also reported. **(D)** Tropoelastin. Tropoelastin is a protein comprising a signal peptide (SP) followed by alternating hydrophobic and cross-linking regions. The protein is functinctinally composed of an N-terminal domain that confers spring-like properties to the molecule and a C-terminal domain recognizing the α_v_β_3_ integrin. The domains are connected by a bridge region that contains sites for the coarcevation process.

The large central domain comprises 15 type III repeats and includes sites interacting with the N-terminal assembly domain as well as binding sites for plasma membrane components. Each type III module contains approximately 90 aa residues and is organized in two β-sheets containing three and four strands, respectively (Main et al., 1992; Dickinson et al., 1994). The central domain of Fn, called cell-binding domain (CBD), contains an arginine-glycine-aspartate (RGD) motif in type III module number 10 (FnIII_10_) that directly binds to the integrin α_5_β_1_ and a synergy site (FnIII_9_) that re-enforces binding of Fn to cell surface. Binding of FnIII_10_ motif to the integrin connects the ECM to the cytoskeleton and generates signaling pathways. The region following the CBD includes a second high affinity heparin-binding domain and the C-terminal fibrin-binding domain (Figure 2A; Maurer et al., 2015).

### Fibrinogen

Fbg is a large, soluble plasma glycoprotein synthesized by hepatocytes. Fbg consists of two monomers, each composed of three non-identical chains, designated Aα, Bβ and γ, linked together by disulfide bonds. Thrombin converts Fbg to fibrin monomer, releasing a 16 residue fibrinopeptide A (FPA) from the Aα chain and a 14 residue fibrinopetide B (FPB) from the Bβ chain. The uncovered N-terminal ends in the α- and β-chains of Fbg promote interactions with complementary sites in the C-terminal domains of neighbouring Fbg molecules, thus triggering the protofibril formation and soft clot. The soft clot is converted to hard clot by activated transglutaminase (FXIIIa), which promotes covalent crosslinks between the α and β chains (Weisel, 2005). The fibrin network is the major protein component of the hemostatic plug. Besides its primary role in clotting, Fbg binds *via* the RGD motifs contained in the α chains to the platelet integrin GPIIb/IIIa and is involved in platelet aggregation and thrombus formation (Figure 2B). Fbg is also deposited on implanted medical devices (Plow et al., 2000).

### Plasminogen

The circulating mature form of Plg is a 92-kDa zymogen known as Glu-Plg that contains a glutamic acid at the N-terminus. Glu-Plg contains a hairpin-loop structure, the PAN domain, followed by five homologous kringle modules (K1–K5), each containing a typical pattern of four cysteine, and a proteinase catalytic domain. The kringle modules interact with fibrin clots and surface receptors from both eukaryotic and bacterial cells. Proteolytic activation of Plg to plasmin occurs through cleavage of the Arg^561^–Val^562^ peptide bond in the catalytic domain by the tissue Plg activator (t-PA) and urokinase Plg activator. The cleavage results in the formation of plasmin, the active enzyme that contains the catalytic triad made up of the amino acid residues His^603^, Asp^646^ and Ser^741^ (Figure 2C). Staphylokinase, a product of a gene located on lysogenic bacteriophages in *S. aureus*, can activate Plg when it forms a 1:1 stoichiometric complex with human plasmin (Sanderson-Smith et al., 2012; Verhamme et al., 2015).

### Elastin

The protein elastin makes up the highly insoluble amorphous component of elastic fibers in the extracellular space of many tissues such as the lung, dermis and large blood vessels. Elastin is composed largely of glycine (33%), proline (10–13%) and other hydrophobic residues (over 40%). Individual polypeptide chains of soluble elastin, named tropoelastin, assemble into large fibers stabilized by multiple lysine-derived cross links (desmosines) formed by the copper-requiring extracellular enzyme lysil oxidase (Mecham, 2018; Kozel and Mecham, 2019). Tropoelastin contains an N-terminal domain that confers spring-like properties to the molecule and a C-terminal region that mediates binding to α_v_β_3_ integrin *via* a GRKRK motif (Bax et al., 2009; Baldock et al., 2011). The central domain contains contact sites for elastin coarcevation, a process observed in specific conditions of temperature, ionic strength and pH and where tropoelastin molecules associate to form elastic fibers (Figure 2D; Dyksterhuis et al., 2007).

### Histones

Histones are basic proteins classified either as lysine-rich (H1, H2A, H2B) or arginine-rich (H3 and H4). In eukaryotes, histones H2A, H2B, H3 and H4 have a structural role in the formation of the nucleosome units, concurring to the assembly and compactation of chromatin. Together with DNA, histones are also the most abundant proteins in neutrophil extracellular traps (NETs), which are networks of extracellular fibers released by neutrophils to catch and kill infecting bacteria (Sollberger et al., 2018). Histones are also released into the bloodstream during severe sepsis (Xu et al., 2009). Histones have potent antimicrobial activity due to properties similar to cationic antimicrobial peptides. Histones H2A, H3 and H4 have been shown to have anti-staphylococcal activity (Morita et al., 2013; Pietrocola et al., 2019).

## Mechanisms of FnBPs Interaction With Ligands

### Ligand Binding to A Domain of FnBPs

**FIGURE 3:**
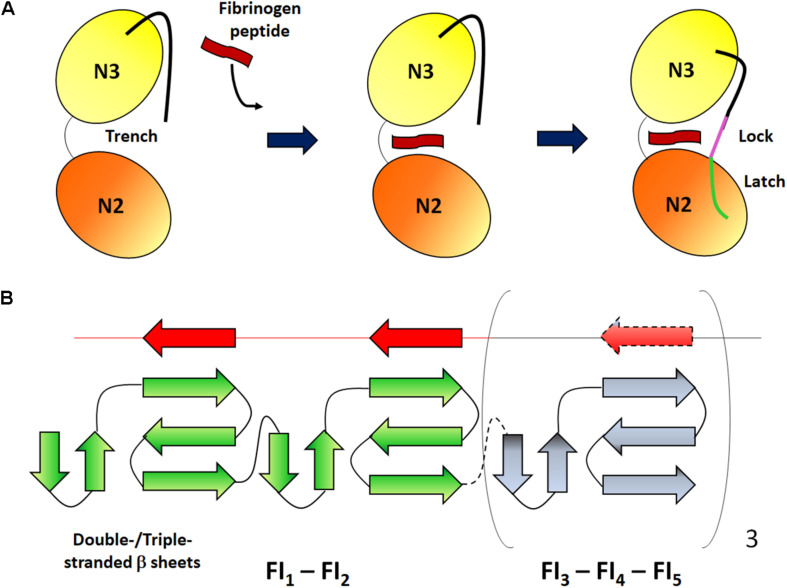
Schematic representation of the binding mechanisms of Fbg and Fn. **(A)** Dock, lock and latch mechanism. The N2 and N3 subdomains of the MSCRAMM form a open trench where a Fbg peptide (red) can be inserted. After ligand binding, the C-terminal unstructured extension of the N3 subdomain undergoes conformational changes so that the segment covers the bound peptide and locks it in place (magenta) and binds to a β-strand in the N2 subdomain (green). Adapted from Geoghegan and Foster, 2017. **(B)** Tandem β-zipper mechanism. The Fn-binding repeats form a disordered region between the A domain and the C-terminal end of FnBPs. Each linear repeat (red) can complex with the type I modules (green) of the N-terminal domain forming additional short β-strands and extending the triple-stranded β-sheet on each module. Adapted from Matthews and Potts (2013).

Sequence homology between FnBPA and clumping factor A (ClfA), a CWA Fbg-binding protein, the observation that the binding site in Fbg is the same (the C-terminal segment of the γ chain) and the finding that both ClfA and FnBPA bind to Fbg with similar affinity, all suggest a very similar mechanism of Fbg binding. Both the proteins interact with Fbg by the DLL (Dock, Lock and Latch) mechanism (Ganesh et al., 2008; Stemberk et al., 2014). In the case of ClfA, binding is initiated by docking of a peptide mimicking the C-terminus of the γ chain (HHLGGAKQAGDV) in the hydrophobic trench formed between the N2 and N3 subdomains. Next, the peptide-MSCRAMM complex induces a reorientation of the C-terminal end of N3 subdomain, so that this segment covers the bound peptide and locks it in place. In the final latching event the short segment inserts within the N2 subdomain and complements a β-sheet in this subdomain (Figure 3A; Deivanayagam et al., 2002). Notably, the A domain of ClfA also binds to a site located at the top of subdomain N3 and far away from the Fbg peptide binding trench (Ganesh et al., 2016). Some subtle differences exist between the interactions of FnBPA or ClfA with Fbg. It seems that binding of Fbg peptide to FnBPA does not require the involvement of latch strand residues and that peptide binding marginally affects the conformation of FnBPA. Moreover, it is not clear whether binding to Fbg by FnBPA involves a two-site mechanism as reported for ClfA (Stemberk et al., 2014).

Combining single molecule atomic force microscopy (AFM) and steered molecular dynamics simulations, Milles et al. (2018) revealed that the surface protein SdrG from *Staphylococcus epidermidis* binds to Fbg with a strength of 2 pN, similar to that of the covalent bonds. These authors also found that this mechanostability is related to an intricate hydrogen-bonding network between SdrG and a target Fbg peptide, which is independent of peptide side chains. A similar side-chain independent mechanical stability was observed with ClfB, another Fbg-binding adhesin from *S. aureus* (Milles et al., 2018). It remains to be determined whether this hydrogen bond mechanism may be extended to ClfA and FnBPs.

The A domain of FnBPA and FnBPB also binds Plg by a mechanism that does not involve DLL (Pietrocola et al., 2016). Differently from the Fbg binding, a single subdomain was required for Plg binding to FnBPs: subdomain N2 for FnBPA and subdomain N3 for FnBPB (Herman-Bausier et al., 2017). Notably, preincubation of FnBPB with Fbg increased by tenfold binding of Plg to adhesin expressed on the bacterial surface, suggesting that the conformational change of FnBPB occurring upon Fbg binding induces a better exposure of the Plg binding site and a higher interaction with the MSCRAMM (Herman-Bausier et al., 2017). Capture of Plg from serum and its conversion to plasmin by host or bacterial activators promotes degradation of opsonins and facilitates spreading of bacteria in infected tissue (Pietrocola et al., 2016).

In addition, FnBPB binds histones, most likely by DLL, with an affinity which is 20-fold higher than for Fbg, indicating that FnBPB will bind preferentially histones when both proteins are incubated with the MSCRAMM. FnBPB could simultaneously bind H3 histone and Plg and activation of bound PLG resulted in cleavage of H3. Thus, FnBPB neutralizes the bactericidal activity of histones by direct capture of histones and prevention of their access to the surface. Alternatively, FnBPB can bind to Plg which, when activated to plasmin, can cleave the sequestered histone. Also FnBPB expression was shown to be responsible for protecting *S. aureus* from the bactericidal effect of the total histones released by neutrophils in the form of NETs (Pietrocola et al., 2019). Finally, the A domain of FnBPB can also bind to Fn by a mechanism that does not involve DLL (Burke et al., 2010).

Moreover, both the A domains of FnBPA and FnBPB promote adhesion of *S. aureus* to tropoelastin (Roche et al., 2004). The A domain of FnBPA binds to tropoelastin with high affinity (Keane et al., 2007a, b). Binding occurs at multiple sites, suggesting that these interactions involve repetitive sequence stretches of tropoelastin. The interaction is stabilized by negative charges on the A domain of FnBPA and lysine residues on tropoelastin (Keane et al., 2007a). The mechanism of elastin binding to FnBPs is unknown.

### Binding of the Repetitive Region of FnBPs to Fn

The region that follows the Fbg-binding domain is composed of intrinsically random coil repeats. When bound to Fn, the Fn-binding repeat region acquires an ordered secondary structure. The stoichiometry of FnBP binding to Fn is estimated to be between six to nine Fn molecules per FnBP and at least six repeats bind to the NTD modules with high affinity (Fröman et al., 1987; Meenan et al., 2007). Each of the high affinity Fn-binding repeats binds to NTD by adding a short anti-parallel strand to β-sheets of sequential Fn type I modules. Thus, the intrinsically disordered Fn-binding repeats adopt a conformation determined by the binding to the NTD type I modules. Interactions between the FnBPs and Fn are also stabilized by hydrophobic and hydrophilic bonds. The tandem array of β-strands is called the β-zipper formation (Figure 3B; Schwarz-Linek et al., 2003).

## Amino Acid Polymorphism in FnBPs and Effects on Fn and Fbg Binding

Seven different *fnbB* allelic variants were identified in both human and bovine isolates. The A domains of the corresponding seven isotypes of the FnBPB are 61–85% identical in sequence and each variant bound to ligands (Fbg, Fn and elastin) with similar affinity (Burke et al., 2010). Likewise, several isotypes of the A domain of FnBPA were identified which are between 66 and 76% identical in amino acid sequence in any pair-wise alignment. The isotypes retain their ligand-binding activity but differ antigenically and exhibit limited immune-cross reactivity (Loughman et al., 2008). Such polymorphisms in FnBPA were confirmed by a comparative study of *S. aureus* strains that originated from humans with infected and uninfected cardiac devices. These studies also found that amino acid substitutions in Fn-binding repeats 5 and 9 of FnBPA isolated from patients with infected cardiac implants confer a higher binding affinity for Fn, suggesting that strains with these substitutions could better impact colonization of damaged tissue or implanted devices (Casillas-Ituarte et al., 2012). Surprisingly, amino acid substitutions in the repeat region of FnBPA also affected binding of FnBPA to Fbg. These results were unexpected since these substitutions are quite distant from the trench binding site in the A domain of FnBPA. The authors suggested that the sequence variation of the C-terminal repetitive sequence might determine its fold back on the A domain so that binding of Fbg to the trench is enhanced (Casillas-Ituarte et al., 2019). Importantly, some substitutions in FnBPA significantly strengthened interactions with Fbg upon applications of a tensile force to the bond (Casillas-Ituarte et al., 2019). This observed strengthening of the Fbg-FnBPA interaction in the presence of a tensile force is reminiscent of catch-bond behavior, a type of non-covalent ligand-receptor interaction that is enhanced by application of a mechanical force in contrast to the traditional slip bonds that detach under increased force (Bartolo et al., 2002).

## The Biological Role of FnBPA/FnBPB in Staphylococcal Infections

### Adhesion and Invasion

FnBPs contribute to the colonization and infection of the host by *S. aureus via* adhesion to Fn present in the extracellular matrix of the tissues. Following adhesion, *S. aureus* is able to invade a variety of non-professional phagocytic mammalian cells, such as epithelial cells (Dziewanowska et al., 1999), fibroblasts (Usui et al., 1992), endothelial cells (Peacock et al., 1999), osteoblasts (Ahmed et al., 2001) and keratinocytes (Mempel et al., 2002). The significance of bacterial invasion is unclear, but it could be involved in escape from immune defences of the host and protection from antibiotics. Fn-bridging between FnBPs and α_5_β_1_ integrin on the host cell surface is sufficient to induce zipper-type uptake of staphylococci (Sinha et al., 1999). A study focussed on the FnBR-dependent role of endothelial cell invasion by *S. aureus* demonstrated that cell invasion can be facilitated by FnBRs with high affinity for Fn, while low affinity FnBRs expressed on the staphylococcal surface mediate adhesion but not the invasion. Furthermore, multiple FnBRs increased the efficiency of invasion without altering the uptake mechanism (Edwards et al., 2010). Small colony variants (SCVs), a subpopulation of *S. aureus* originating by mutations in metabolic genes and adapted to persist viable in the intracellular milieu (Sendi and Proctor, 2009), express high levels of FnBPs, a property which facilitates invasion of the host cells (Vaudaux et al., 2002). Recently, a model for invasion involving FnBPA, Fn and the α_5_β_1_ integrin has been proposed. The dimeric native Fn in solution predominantly exists as a compact globular conformer where the modules ^9^FnIII-^10^FnIII located in the central cell-binding domain are occluded and impeded by binding to α_5_β_1_ integrin molecules on the surface of cells. Upon binding to the NTD of Fn, FnBPA repeats disrupt specific intramolecular contacts within Fn chains resulting in exposure of the cryptic α_5_β_1_ binding sites and facilitation of Fn interaction with and clustering of integrins (Liang et al., 2016). Clustering of integrins promotes a recruitment of proteins such as vinculin, zyxin and tensin, as well as activation of focal adhesion kinases (FAKs) and Src to the site of bacterial attachment. The concerted activity of FAK and Src results in tyrosine phosphorylation of several effectors including cortactin, actin polymerization and the final bacterial invasion (Hauck and Ohlsen, 2006). This model of invasion for FnBPs is reminiscent of the afimbrial adhesin YadA from *Yersinia pseudotuberculosis* (Heise and Dersch, 2006) and the surface protein F1 from *Streptococcus pyogenes* which promote host cell invasion *via* Fn-bridging to integrin α_5_β_1_ (Ozeri et al., 1998). Together these findings support the concept that FnBP-mediated invasion mechanism is a pretty common process among bacteria.

### FnBPs in Platelet Activation/Aggregation and Thrombus Formation

Binding of *S. aureus* to platelets is a critical factor in the development of infective endocarditis. The process occurs in two stages. Firstly, staphylococci interact with quiescent platelets and promote intracellular signaling *via* the activation of the integrin GPIIb/IIIa. Secondly, activated platelets aggregate and contribute to the formation of thrombi and vegetations on the surface of heart valves. The FnBPs expressed on the surface of *S. aureus* cells from exponential phase of growth are the dominant platelet activating factors. In a study performed with *S. aureus* or *L. lactis* expressing either the Fbg-binding domain or the repetitive Fn-binding domain, Fitzgerald et al. (2006) demonstrated that each region promoted platelet activation, suggesting that FnBPA possesses two different mechanisms to activate resting platelets. Fbg and Fn present in the blood independently bind to the A domain and the repetitive region of FnBPA, respectively, and cross-link the bacterial surface to the low affinity GPIIb/IIIa and trigger activation and subsequent aggregation of the resting platelets. Furthermore, specific antibodies bound to FnBP domains act as additional, essential bridges between the FnBPs and the platelet immunoglobulin Fc receptor FcγRIIa (Fitzgerald et al., 2006). ClfA, another important Fbg-binding protein, expressed by stationary phase cells cooperates to activate and aggregate platelets in a fashion similar to FnBPs (Loughman et al., 2005). Other CWA proteins such as ClfB, SdrE and protein A have also been shown to have a role in the process (O’Brien et al., 2002).

### FnBPs Engagement in Homophilic Interaction and Biofilm Formation

FnBPA and FnBPB expressed by clinically relevant HA-MRSA strains promote biofilm development (O’Neill et al., 2008; Vergara-Irigaray et al., 2009). FnBPs are thought to induce biofilm formation by a mechanism based on multiple, Zn^2+^-dependent, hemophilic, low affinity bonds between the A domains of FnBPA or FnBPB located on adjacent cells. By using atomic force microscopy techniques it was also demonstrated that the mean adhesin force for FnBPA-FnBPA interactions is 190 pN, which is approximately 10 times lower than the binding that occurs between Fbg and its adhesin (2000 pN binding force). The weak force needed to separate neighbouring cells might help cell detachment during the dispersal phase that follows the biofilm formation (Herman-Bausier et al., 2015).

## Immunological Properties of FnBPA/FnBPB

In view of the important role played by FnBPs in pathogenesis it is crucial to define the immunological properties and the potential of these virulence factors in vaccine development. A study performed by Zuo et al. (2014) demonstrated that A domain had a strong immunogenicity and induced an increased survival of mice immunized with the antigen, while the C-terminal Fn-binding region had poor antigenic properties. Moreover, an amino acid segment located between the subdomain N1 and N2 showed immunogenicity and vaccine potential comparable to the full length A domain of FnBPA (Zuo et al., 2014). More specifically, a linear B cell-epitope (IETFNKANNRFSH) in the same subregion of FnBPA was identified and shown to evoke a protective immune response against *S. aureus* infection in immunized mice (Ma et al., 2018). Thus, region A of FnBPs seems to be a promising component for a staphylococcal vaccine.

Reports regarding the immunogenicity of the Fn-binding region of FnBPs are controversial. Immunization of rats with the repeat region of FnBPA induced protection against endocarditis (Rennermalm et al., 2001). Conversely, FnBRs weakly reacted with IgG isolated from sera of patients with staphylococcal endocarditis or region-specific monoclonal antibodies (mAbs) in the absence of Fn, but reacted more strongly in the presence of Fn. Thus, patients’antibodies and mAbs recognize Ligand-Induced Binding Site (LIBS) neo-epitopes formed upon FnBR binding to Fn. Notably, in the presence of Fn high affinity repeats exhibited a better reactivity than low affinity repeats with IgG from patients’sera, suggesting that during *S. aureus* infections patients develop preferentially LIBS antibodies to high affinity FnBRs (Rindi et al., 2006; Meenan et al., 2007). Importantly, LIBS antibodies did not show any antigen neutralizing activity. This observation should be taken into due consideration in the development of an anti-staphylococcal vaccine.

## FnBP-Like Proteins From Other Staphylococcal Species

*Staphylococcus pseudintermedius*, a staphylococcal species responsible of canine otitis, pyoderma and surgical wound infections, expresses two cell wall-anchored proteins, SpsD and SpsL, showing a organization and functionality similar to FnBPA and FnBPB and that are likely to be important in tissue colonization and pathogenesis (Geoghegan et al., 2009; Bannoehr et al., 2012; Richards et al., 2018). SpsD contains a secretory signal sequence at the N-terminus and a C-terminal LPXTG motif required for anchoring the protein to the wall peptidoglycan. The N-terminal A domain, consists of three subdomains N1-N3 and is 40% identical to the A domain of FnBPB of *S. aureus*. Located distal to the A domain are a connecting region (C region) and a repeat region (R region). The N-terminal A domain of SpsD binds Fbg, Fn, elastin and cytokeratin 10. The binding site of the SpsD A region was mapped to residues 395–411 in the fibrinogen γ-chain. SpsD also bound to Gly- and Ser-rich omega loops within the C-terminal tail region of cytokeratin 10 (Pietrocola et al., 2013).

SpsL contains a signal sequence at the N-terminus followed by an A domain organized in three subdomains N1, N2, and N3 and a domain made up of multiple, tandemly identical repeats showing weak homology to the repetitive units of FnBPs of *S. aureus* (Pietrocola et al., 2015). Binding to Fbg occurs *via* the N2-N3 subdomains by a mechanism analogous to the DLL mechanism. There are multiple binding sites in the fibrinogen α-chain C-domain (Pickering et al., 2019). The connecting region of SpsD and the repetitive domain of SpsL represent the main binding sites of Fn binding and are involved in the cell invasion (Pietrocola et al., 2015). The use of LIBS antibodies raised against FnBPA has provided cues to identify in both these regions of SpsD and SpsL minimal binding units sequentially and structurally related to the repeated motifs of FnBPA of *S. aureus* (Viela et al., 2020). This observation can pave the way to get insights on the organization of these regions and for designing peptide analogs with inhibitory potential on MSCRAMM-Fn interaction. Recently, a surface protein from *S. delphini* called *S. delphini* surface protein Y (SdsY) has been described. SdsY shares 68% identity with SpsD from *S. pseudintermedius*, 36–39% identity with FnBPs and possesses the typical structure of Fn-binding proteins, including an N-terminal signal sequence, an A domain, a typical repeated region and a cell wall anchor motif. SdsY is involved in Fn-dependent internalization of bacteria into host osteoblast cell lines (Maali et al., 2020).

The *in silico* screening approach permitted identification of FnBP-like proteins in *S. argenteus* and *S. schweitzeri*, two species closely related to *S. aureus* (Weinman et al., 2020). Structural and functional studies on these proteins are lacking.

## FnBPs as Virulence Factors

The role of FnBPs in virulence has been the object of several studies over the years. Since FnBPs are multifunctional, it is reasonable that these proteins play different roles in specific pathogenetic contexts. Importantly, the interpretation of these *in vivo* investigations should be taken with caution due to the number of factors (bacterial strain used, route of infection and infection model) on which the outcome of the infective process depends.

### Role of FnBPs in Virulence of *S. aureus* Skin Abscess Infection

Using mouse models of skin abscess formation, it was shown that a *S. aureus* strain lacking all of its cell-wall anchored proteins was less virulent that its wild type strain. Strains specifically lacking FnBPA and FnBPB or other surface CWA protein such as protein A and ClfA also showed a significantly reduced virulence. Conversely, when a model of skin necrosis was studied, the *S. aureus* surface proteins could not be shown to be involved (Kwiecinski et al., 2014). *S. aureus* binds also to skin sections of human patients with atopic dermatitis (AD) (eczema) more efficiently than the biopsies taken from normal or psoriatic skins. In human atopic skin enhanced bacterial binding was primarily due to the increased level and availability of fibronectin present in the stratum corneum and in these conditions FnBPA and FnBPB expressed on the surface of *S. aureus* promoted better adhesion to AD sections compared to healthy ones (Cho et al., 2001).

### Involvement of FnBPs in Infective Endocarditis

The role of *S. aureus* ClfA and FnBPA in the colonization of damaged valves in rat with experimental endocarditis was studied by Que et al. (2005) FnBPA-positive lactococci showed increasing bacterial concentrations in vegetations and spleens. The transformants also invaded the adjacent endothelium, possibly due to their capacity to trigger cell invasion. On the contrary, ClfA-positive lactococci were restricted to the vegetations. The authors also investigated the separate roles of the A and the repetitive domains of FnBPA and found that deletion of the A domain did not alter Fn binding and cell invasion, while it eliminated the colonization and infection of the valves *in vivo*. Furthermore, when lactococci expressing FnBPA deleted of the Fbg-binding domain were supplemented *in cis* or *in trans* with the Fbg-binding activity of ClfA acquired the A domain-associated functionality *in vitro* and *in vivo*. Summing up, the Fbg- and Fn- binding actions of the A and repetitive domains of FnBPA could synergically cooperate in the colonization of valves and invasion of endothelium (Que et al., 2005).

### FnBPs and Their Role in Bacteremia and Sepsis

Bacteremia due to *S. aureus* can lead to the development of sepsis, a general inflammatory response to infection including fever, weakness, rapid heart rate and breathing rate. The response affects many internal organs such as the kidneys, heart, and lungs. Central venous catheters, surgically implanted materials, and orthopedic prostheses are the most important risk factor for bacteremia and invasive infection caused by *S. aureus* (Jensen et al., 1999) and these prosthetic devices help gain access to the bloodstream (Musher et al., 1994). To spread in the tissues and induce abscess lesions, *S. aureus* needs to leave the vasculature. FnBPA and FnBPB significantly contribute to host cell adherence *in vitro* and *in vivo* (Peacock et al., 1999; Kerdudou et al., 2006) and invasion of endothelial cells (Sinha et al., 1999). Moreover, *S. aureus* invasion of host cells and virulence in sepsis is facilitated by expression of multiple repeats in FnBPA (Edwards et al., 2010). It is still unclear how staphylococci exit endothelial cells, cross the basement membrane and reach tissues. Possibly, toxins and secreted bacterial proteases play a role at this stage. Importantly, cooperation between FnBPA and FnBPB is indispensable for the induction of severe infection resulting in septic death (Shinji et al., 2011).

### Staphylococcal Pneumonia and FnBPs

Until recently, pneumonia caused by *S. aureus* has been regarded as a secondary effect of viral respiratory infections. Now, staphylococcal pneumonia has emerged as an important clinical problem. Several virulence factors have gained attention as potential causative virulence agents of the disease, among others Panton Valentin leukocidin (PVL) (Labandeira-Rey et al., 2007) and the collagen-binding adhesin Cna (de Bentzmann et al., 2004; Otsuka et al., 2006). Using a humanized tracheal xenograft model in the nude mouse and cultures of HAEC (human airway epithelial cells) it has been shown that FnBPs have an important role in the colonization of the airways by *S. aureus* and a potential contributory role in the onset of staphylococcal pneumonia (Mongodin et al., 2002).

### Foreign Body Infections and FnBPs

*S. aureus* is one of the most important causes of implant-associated infections (Arciola et al., 2018). The success of the pathogen in this context depends on rapid and efficient adhesion to virtually all biomaterial surfaces and biofilm development. Staphylococcal attachment to and colonization of the biomedical implants is largely due to the arsenal of surface adhesins expressed by this bacterium and to the rapid coating of the implants with proteins from blood and interstitial fluids, mostly Fbg and Fn (Speziale et al., 2009; Franz et al., 2011). Thus, one should expect that *S. aureus* strains, the majority of which are equipped with FnBPs, will find ideal conditions for adhesion to Fn- and Fbg-coated biomedical implants. Expression of FnBPA and FnBPB represents an additional critical element for the formation of a proteinaceous biofilm, persistence of implant infections and bacterial dissemination to other body sites.

The prompt integration of biomaterials into resident cells in nearby tissues is crucial for the success of implantation of many medical devices (Gristina, 1987) and for preventing bacterial adhesion to abiotic surfaces or biotically coated surfaces (Stones and Krachler, 2016). However, considering the ability of *S. aureus* to invade endothelial cells and osteoblasts, even tissue-integrated biomaterials could become the target for the staphylococcal infection. Thus, biofilm formation and ability to invade host cells, both mediated by FnBPs shelter *S. aureus* cells and encourage persistence of infection in medical devices.

## Conclusion

In this review we summarize the most recent findings on the structure and function of FnBPs from *S. aureus*.

If structural biology has revealed the mechanisms of FnBPA and FnBPB interacting with Fbg and Fn (DLL and β-zipper mechanisms), the definition of the X-ray crystal structure of FnBPs in complex with the new ligands such as Plg and histones and the binding mechanisms remain to be revealed. Moreover, a more detailed structural analysis of homophilic interactions will be beneficial for understanding the biochemical basis of biofilm formation promoted by FnBPs. These studies would help in designing inhibitors of the various processes mediated by these MSCRAMMs and in controlling infection. Furthermore, it will be of biological importance to test the role of Plg binding to FnBPA and FnBPB in infection models with mice.

However, one should be cautious when studies with murine infection models are performed. In fact, differently from human Plg, the mouse Plg reacts poorly with staphylokinase (Peetermans et al., 2014). Moreover, it should be experimentally established whether FnBPs recognize murine Plg efficiently. Thus, for a full investigation of the role of Plg binding and activation in infection it is worth exploring transgenic mice expressing human Plg.

FnBPA and FnBPB share several structural and functional similarities. Hence, an important question is why *S. aureus* cells express two FnBPs with apparently similar properties. It is possible that expression of each adhesin is differentially regulated and dependent on external factors such as growth conditions. Indeed, a closer examination of these proteins reveals subtle differences both in the structure and function. For example, both proteins bind to Plg *via* different subdomains of region A, but, more intriguingly, only FnBPB protein interact with histones and prevents them from damaging the bacterial membrane. The discovery of new ligands and functions for FnBPs also deserves attention. Such discoveries must be followed by a detailed biochemical and physical investigation and rigorous analysis *in vivo* to prove the biological significance of bacterial interactions with new ligands. Another issue regards the immunological properties of FnBPs. The repetitive region of FnBPs does not appear to be promising for vaccine development, while it remains to be determined whether A region of FnBPs can be used as vaccine. In fact, if some observations indicate that FnBPA antibodies are protective in animal models of infection (Zuo et al., 2014), on the other side, the A region shows a significant polymorphism and antibodies raised against one isoform react weakly with the others (Loughman et al., 2008; Burke et al., 2010). Several CWA proteins including FnBPs are released in the growth medium by the action of endogenous proteases (McGavin et al., 1997) or by mutational events (Grundmeier et al., 2004) and possibly they are involved in important biological functions, for example, modulation of MSCRAMM binding to ligands and interference with events such as cell invasion, biofilm formation and immune evasion (O’Halloran et al., 2015). Therefore, it is worth exploring the role of soluble FnBPs in the regulation of the multivalent activity of these proteins. Finally, since FnBPs are multifactorial and involved in different pathologies, a more clear definition of the role played by these proteins in specific infective processes is key for the development of novel therapeutic approaches to control the bacterium in diverse disease settings.

## Author Contributions

PS conceptualized and coordinated the work. PS and GP contributed to this article in terms of data collection and writing and performed critical revision for the work. Both authors contributed to the article and approved the submitted version.

## Conflict of Interest

The authors declare that the research was conducted in the absence of any commercial or financial relationships that could be construed as a potential conflict of interest.
